# Selective Transfer Hydrogenation of Furfural to Isopropyl Levulinate: An In Situ One‐Pot Cascade Approach

**DOI:** 10.1002/cssc.202502378

**Published:** 2026-03-01

**Authors:** Saravanan Subramaniyan, Christian Hering‐Junghans, Eszter Baráth

**Affiliations:** ^1^ Leibniz‐Institut für Katalyse e.V. (LIKAT) Rostock Germany

**Keywords:** furfural, homogenous catalysis, levulinates, ruthenium, transfer hydrogenation

## Abstract

Within the current article, we were interested in describing the complete conversion of furfural (FF) as a green starting material to small oxygenates using isopropanol (IPA) as a solvent and as a H‐donor. We applied a series of Ru‐based complexes, and found that the most active Ru‐complex in terms of isopropyl levulinate (IPL) formation at 130°C after 21 h was Ru‐MACHO‐BH (Ru‐MACHO‐BH = carbonylhydrido[2‐(diphenylphosphino‐*κP*)‐N‐[2‐(diphenylphosphino‐*κP*)ethyl]ethanamine‐*κN*][tetrahydridoborato(1‐)‐*κH*]ruthenium(II)). We used two catalytic functions, one was the metal function to cover the hydrogenation of the aldehyde by Ru, and a Brønsted acidic function to secure the specific sub‐steps of the reaction network (e.g.: hydrolysis of furfuryl alcohol as an intermediate, hydrolysis of 2‐(isopropoxymethyl)furan (FE) to IPL). We observed that, the harmonization of these two functions leads to a highly selective reaction toward IPL (complete conversion, 67% yield). Furthermore, the reaction cascade can be directed until the final product, γ‐valerolactone (GVL) (15% yield). The Brønsted acid−Ru‐MACHO‐BH catalyst system provides a unique reaction media where the usual humin/high molecular weight side products formation can be suppressed, while the nature of catalysis stays intact (homogeneous during the entire reaction coordinate).

## Graphical abstract

In this article, we applied a combination of metal (0.5 mol%) and acid (1 mol%) that functioned harmoniously to selectively convert a bio‐based starting material, furfural, into small oxygenates. We used a transfer hydrogenation reaction with isopropanol acting as both the reducing agent and the reaction medium. Isopropyl levulinate, a key intermediate in the chemical industry, was isolated as the major product with a yield of 67%.

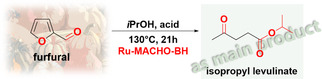

## 1 Introduction

**FIGURE 1:**
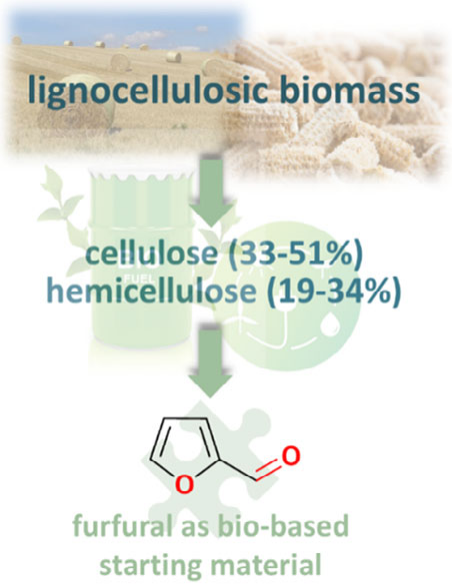
Lignocellulosic biomass as source for the synthesis of key intermediates, small oxygenates, such as furfural.

The continuous depletion of fossil resources, such as crude oil and natural gas [1, 2], necessitates the development of new strategies to meet global fuel and chemical demands. The transition from fossil, nonrenewable, resources to bio‐based, potentially recyclable, chemical feedstocks is essential for ensuring a more sustainable future and for decreasing CO_2_ emissions [3]. Biomass, particularly lignocellulose, is a readily available and renewable feedstock for the production of platform chemicals in sustainable fashion [4]. Among these, furfural (FF) (Figure 1) and other furan derivatives are of particular interest, as they bridge the gap between biomass feedstocks and the chemical industry [5].

**FIGURE 2:**
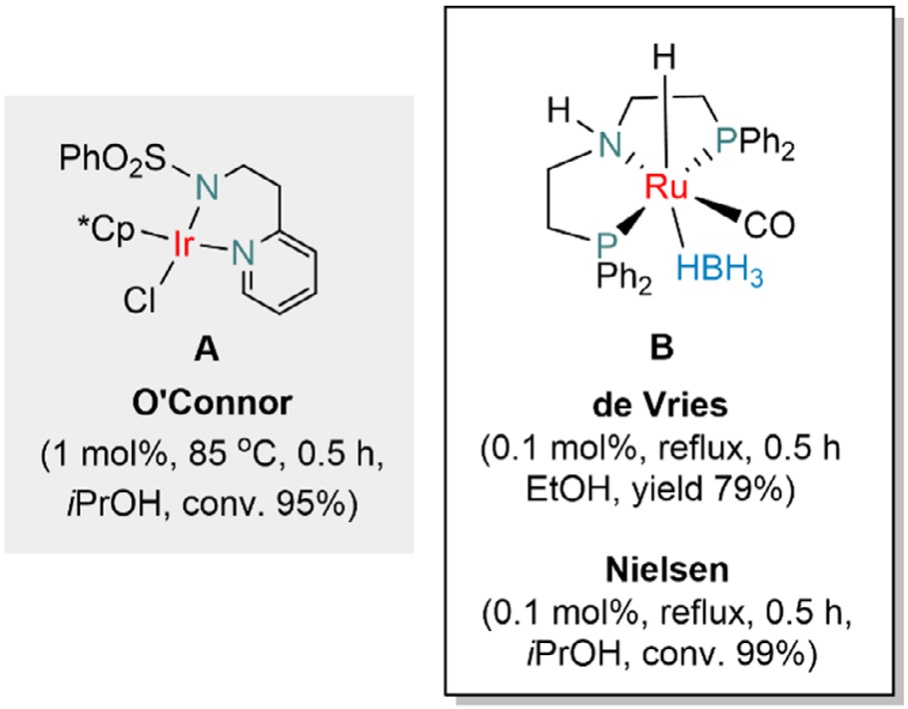
Reported Ir (**A**) [15] and Ru (**B**) [16, 17] complexes for the transfer hydrogenation of FF toward FA.

FF is considered as one of the most promising bio‐based platform molecules. It is derived from the pentoses (C5 sugars) contained in hemicellulose through an acid‐catalyzed dehydration reaction [6, 7]. FF can be further transformed into many value‐added materials through processes such as hydrogenation, oxidation, and cyanation to yield furfuryl alcohol (FA), furoic acid, or 2‐furonitrile, respectively [8]. Among these, homogeneous and heterogeneous transition metal catalyzed hydrogenations of furfural have emerged as a major pathway toward important platform chemicals [9, 10]. However, the use of potentially hazardous hydrogen gas (H_2_) and the risks associated working with high‐pressure apparatuses necessitate the use of alternative H_2_ sources [11]. Instead, readily available and inexpensive alternatives, such as alcohols, can be utilized for transfer hydrogenation (TH) reactions [12, 13]. There are only a limited number of examples in homogeneous catalysis using TH to convert FF into value‐added chemicals [14, 15, 16, 17]. For instance in 2017, O’Connor reported the TH of FF with *i*PrOH (IPA) as a hydrogen donor without any additive by using Ir(III) half–sandwich complex **A** (Figure 2) at 85°C for 30 min with a loading of **A** of 0.5 mol% to produce FA in high yields [15]. (In 2018 Shimazu and coworkers reported using La_2_O_3_ (as heterogeneous catalyst) in alcohol solvents for the TH of aldehydes and ketones into the corresponding alcohols [18]).

Efficient and selective hydrogenation and TH can be performed using P, N, P pincer complexes [19]. When reviewing the literature specifically Ru, Rh, Ir pincer complexes have been used for the reduction of furanic compounds in TH protocols [20]. In the majority of recent publications on the hydrogenation of carbonyl compounds and dehydrogenation of alcohols Ru‐pincer complexes have been utilized [21]. Notably, in 2018, De Vries and coworkers employed the commercially available P, N, P Ru pincer complex **B** (Ru‐MACHO‐BH = carbonylhydrido[2‐(diphenylphosphino‐*κP*)‐N‐[2‐(diphenylphosphino‐*κP*)ethyl]ethanamine‐*κN*][tetrahydrido‐borato(1‐)‐*κH*]ruthenium(II)) (Figure 2), at a catalyst loading of 0.1 mol% for the selective TH of FF to FA using *i*PrOH as a solvent with 79% isolated yield [16]. Later, in 2022, Nielsen and coworkers utilized 0.1 mol% of **B**, using EtOH or *i*PrOH as a hydrogen donor at 130°C resulting in nearly quantitative conversion of FF toward FA (Figure 2) [17]. In 2024, the same group reported the direct hydrogenation (using molecular hydrogen) of FF to γ‐valerolactone (GVL) using Ru‐MACHO‐BH as a precatalyst in EtOH and H_3_PO_4_ as a Brønsted acid additive [22].

**FIGURE 3:**
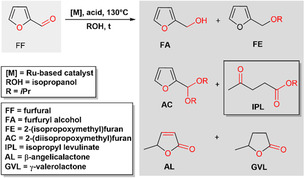
Product spectrum of the catalytic transfer hydrogenation of furfural in the presence of IPA using homogeneous bifunctional catalysis [22] (metal and Brønsted acid function).

As outlined in Figure 3, the selective combination of hydrogenation and acid hydrolysis in a cascade reaction enables the efficient preparation of GVL, however, the selective formation of the intermediate levulinic ester under mild conditions is rather challenging [23]. Alkyl levulinates are valuable products derived from lignocellulosic biomass and represent a promising alternative to levulinic acid in a variety of applications [24]. Among these esters, isopropyl levulinate (IPL) is a promising platform chemical [25] used in biofuels, as a solvent, and/or as building block in polymer chemistry [25, 26]. Additionally, based on its special functional structure IPL serves as a starting material to produce GVL [27], 2‐methyltetrahydrofuran, alkyl valerates, 1,4‐pentanediol, and pyrrolidinones [28].

The production of IPL from FF has been explored by several researchers mainly using heterogenous catalysts [29, 30, 31, 32, 33, 34, 35, 36]. In the following, some specific examples are discussed. Yamanka reported the production of IPL in a stepwise protocol utilizing Ni_3_Sn_2_ (under H_2_) and montmorillonite K10 (under N_2_) in *i*PrOH as a solvent at 120°C for overall 38 h with 48% IPL yield [37]. Another two‐step protocol for the conversion of FF to IPL has been described by Galleti and coworkers, using Cu/Fe_3_O_4_ as a catalyst for the microwave‐assisted FF to FA TH, and in a second step Amberlyst 70 sulfonic resins for the conversion of FA to IPL in moderate overall yields (57%) [36]. By contrast, Zhang demonstrated that regenerated ZrO_2_ with a reduced Lewis acidity supported on mesoporous silica (SBA‐15, calcinated at 550°C), significantly increased the IPL yield to 87% using furfural as substrate, at 160°C for 18 h [38]. More recently in 2021, Feng reported the use of phospotungstic acid (3.5 mol%) as a catalyst with *i*PrOH as a TH agent at 160°C for 7 h, yielding 34% of IPL [39]. Recently, Lu(OTf)_3_ was shown as a catalyst for the TH of FF to IPL using *i*PrOH in a homogenous system, giving IPL in 53% yield, albeit considerable humin formation was observed [40].

Heterogeneous catalysts are appealing for their ease of handling and operation. However, challenges such as limited selectivity, reduced activity, and the need for harsh reaction conditions hinder the creation of sustainable processes [41]. Homogeneous catalysis offers distinct advantages over heterogeneous catalysis, particularly due to its well‐defined and purposefully designed catalyst structures, which generally enable higher activities and selectivity.

Herein, we report a cascade one‐pot protocol to selectively convert FF to IPL using Ru‐MACHO‐BH as a precatalyst in homogenous *i*PrOH solutions under acidic conditions (Figure 3).

## 2 Results and Discussion

**FIGURE 4:**
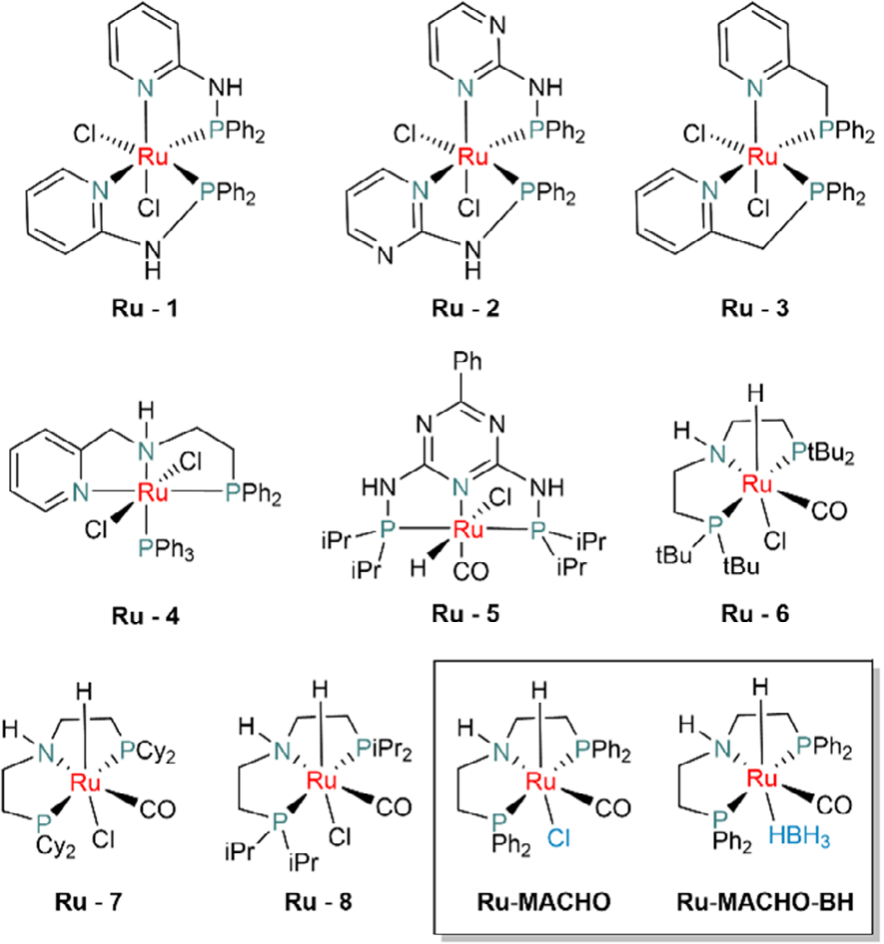
Different ruthenium pincer complexes tested in the TH reaction of furfural.

To commence our studies on the selective transformation of FF toward IPL, we first tested Ru(II) complexes bearing P, N and P, N, N type ligands. Ru(II) bis‐P, N complexes (**Ru‐1**–**Ru‐3**) have been shown to catalyze the TH of FF toward FA using formic acid as a hydrogen donor (please see ESI, Table S1, Figure S1) [42]. Additionally, the ester hydrogenation catalyst **Ru‐4** was tested under basic conditions using *i*PrOH as solvent and H_2_ donor [43]. In all cases, only the formation of the corresponding diacetal (Figure 3, AC) was noted and consequently different precatalysts were tested (Table S1). Next, well‐known alcohol dehydrogenation precatalysts P, N, P‐type Ru(II) complexes **Ru‐5**–**Ru‐8** [44, 45], Ru‐MACHO and Ru‐MACHO‐BH were tested (Figure 4) [46]. Considering the cascade nature of the FF to IPL transformation, methanesulfonic acid (MSA) was added to facilitate the ring‐opening from FA to IPL. While the Ru(II) chloride precursors were activated by the addition of KOH as a base before adding FF and MSA, Ru‐MACHO‐BH was tested only in the presence of MSA. The precatalyst was employed at 0.5 mol% in neat IPA at 130°C, in case of the chloride complexes 0.5 mol% of the base were used and 1.5 mol% of MSA (relating to a net acid loading of 1 mol%). Ru‐MACHO‐BH was used under the same conditions with 1 mol% of MSA as an additive. While **Ru‐5**–**Ru‐7** showed moderate conversion of FF, nearly no IPL was formed, with a black reaction mixture indicating the formation of humins [47, 48]. **Ru‐8** yielded IPL in 15%, indicating a higher activity, however, humin formation could not be suppressed.

Ru‐MACHO with Ph_2_P donor functions yielded 30% of IPL, while also 14% of the corresponding furfuryl *iso*‐propyl ether (FE) were detected. The best activity and selectivity toward IPL were achieved with Ru‐MACHO‐BH (Table 1, entry 6), giving almost quantitative conversion and 50% of IPL. Beside IPL, angelica lactone (AL) and γ‐valerolactone (GVL) were detected, indicating that IPL is an intermediate on the path to GVL. Increasing the precatalyst loading to 1 mol% did not result in any significant increase in conversion or in yield of the products (e.g.: 52% yield of IPL).

**TABLE 1:** Transfer hydrogenation of furfural to small oxygenates using Ru complexes and MSA as Brønsted acid.

Entry	Catalyst	Conversion, %	Yield, %				
			FA	FE	IPL	AL	GVL
**1**	Ru‐5	26	—	1	3	—	—
**2**	Ru‐6	45	—	1	4	—	—
**3**	Ru‐7	34	—	2	6	—	—
**4**	Ru‐8	52	—	6	15	1	—
**5**	Ru‐MACHO	71	1	14	30	3	—
**6**	Ru‐MACHO‐BH^a^	99	—	9	50	3	15
**7**	Ru‐MACHO‐BH^b^	98	98	—	—	—	—

*Note:* Reaction condition: furfural 2 mmol, 5 mL IPA, 0.5 mol% cat. 0.006 M/1.5 mol% (MSA), 0.002 M/0.5mol% KOH was used for catalyst pre‐activation in IPA for 10 min at room temperature, 130°C, 18 h.

a No pre‐activation of the catalyst was performed.

b Without acid, after 30 min reaction time. (Product identification/conversion (%)/yield (%) were determined by GC(‐MS) analysis using dodecane as internal standard, for detailed description please see ESI, Tables S2 and S3, Figures S2–S8).

Notably, Ru‐MACHO‐BH is further hydrogenating AL and yielding 15% GVL. This indicates a higher stability of Ru‐MACHO‐BH under acidic conditions compared to Ru‐MACHO, therefore facilitating a second TH step.

Based on the promising results with Ru‐MACHO‐BH, we further studied the conversion of furfural under the same reaction conditions (Ru‐MACHO‐BH/0.5 mol%, 130°C, IPA as solvent) with different acids at varying concentrations as potential proton donors to carry out the intermediate acid‐catalyzed hydrolysis/ring‐opening from FA(FE) to LA(IPL) (Table 2). Using hexafluoro‐*iso*‐propanol (HFIP) as an acid additive (1 mol%) resulted in full conversion of FF with FA as the major product (Table 2 entry 1). However, performing the reaction in neat HFIP or trifluoroethanol (TFE) as a solvent, resulted in low conversion of FF to FA (Table 2, entries 13 and 14), indicating a drastic decrease in the catalytic performance of the Ru‐complex and a strong dependence on the amount of the acid. Using trifluoracetic acid (TFA) as an additive, 61% conversion with 30% yield of FA beside trace amounts of IPL and AL were measured. Decreasing the acid concentration of MSA to 0.5 mol% suppresses the formation of IPL significantly (2%) (Table 2, entry 9). The presence of FA (51%) and FE (28%) indicate that the ring opening of FA and FE is not achieved at lower MSA concentrations. When using H_2_SO_4_ (1 mol%), 99% conversion was noted, with moderate selectivity towards IPL (47%) and GVL (13%) (Table 2, entry 10). With *p*‐toluenesulfonic acid (PTSA) full conversion was noted, however, the main product in this case was FA beside minimal amounts of FE (Table 2, entry 2). As previously noted, MSA (1 mol%) (Table 2, entry 4), promisingly gives 98% conversion with 59% yield in IPL. HCOOH and H_3_PO_4_ resulted in full conversion, however, these reactions nearly quantitatively gave FA, rather than IPL (Table 2, entries 6 and 8). This suggests that the acid strength plays an intricate role in the outcome of the reaction. Excess amount of H_3_PO_4_ (285.2 mol%) converted furfural 57% with 27% of AL as the main product (Table 2, entry 7), clearly indicating that under strongly acidic conditions IPL is readily ring‐closed to give AL which undergoes a second TH‐step to give GVL.

**TABLE 2:** Transfer hydrogenation of furfural using different Brønsted acids and Ru‐MACHO‐BH.

Entry	Acid	Conversion, %	Yield, %					
			AC	FA	FE	IPL	AL	GVL
**1**	HFIP	99	—	98	—	1	—	—
**2**	PTSA	99	—	44	11	3	—	—
**3**	MSA^a^	99	—	—	9	50	3	15
**4**	MSA	98	—	—	18	59	5	10
**5**	TFA	61	7	30	—	<1	1	—
**6**	H_3_PO_4_	99	2	97	—	<1	2	—
**7**	H_3_PO_4_ b	57	—	9	—	1	27	1
**8**	HCOOH^c^	98	—	95	—	—	—	—
**9**	MSA^d^	99	—	51	28	2	1	—
**10**	H_2_SO_4_	99	—	—	—	47	2	13
**11**	MSA^e^	99	—	—	12	67	6	15
**12**	MSA^f^	93	—	—	5	61 (53)	5	7
**13** g		7	—	5	—	—	—	—
**14** h		10	—	8	—	—	—	—

*Note:* Reaction condition: 18 h, furfural 2 mmol, 5 mL IPA, 0.5 mol% Ru‐MACHO‐BH, 1 mol% of acid.

a 1.5 mol% acid (for comparison from Table 1).

b 285.2 mol%.

c 4 M.

d 0.5 mol%.

e Product distribution after 21 h reaction time.

f Higher scale experiment using 6 mmol of furfural (53% represents isolated yield of IPL).

g HFIP as a solvent.

h TFE as a solvent. (Product identification/conversion (%)/yield (%) were determined by GC(‐MS) analysis using dodecane as internal standard, for detailed description please see ESI, Tables S2 and S3, Figures S9–S13).

Based on the dataset summarized in Table 2, the harmonization between the two catalytic functions (TH (metal catalysis)/hydrolysis (Brønsted acid catalysis)) is a key factor to control the final selectivity pattern of the reaction. Such a fine borderline can be observed when using 0.5 mol%/1 mol%/ and 1.5 mol% of MSA (Table 2, entry 9, 4, and 3). The higher the amount of the acid, the selectivity toward IPL decreases, and more GVL is produced (Table 2, entry 3 and 4). It is important to note that, as a very typical side reaction, the formation of black gum‐type higher molecular weight products (humins) was observed in some cases within our study as well (Table 2, entry 2, 3, 5, 7, 9).

Since 1 mol% of MSA turned out to be the optimal acid amount, further optimizations were carried out. First, the influence of the reaction temperature was explored. At 150°C full conversion of FF was achieved, however, the yield of IPL (45%) dropped considerably with traces of GVL and AL also present (Tables S1–S3). We also carried out the reaction at 110°C, at this lower temperature complete conversion was measured with FA (75% yield) being the main product, besides FE (15%) and minimal amounts of IPL (5%) (Table S3). Additionally, we tested the influence of concentration by lowering the amount of IPA to 3 mL, which at otherwise identical conditions resulted in a drop of IPL‐formation to 40% (Table S4, entry 3), while FF was not fully converted. When conducting the experiment in a 10 mL pressure tube (rather than using a 25 mL tube) in 3 mL IPA the conversion approached 100% while IPL was produced in 59% (Table S4, entry 11), clearly showing that running the reaction at FF concentration of 0.4 M FF in IPA is optimal.

**FIGURE 5:**
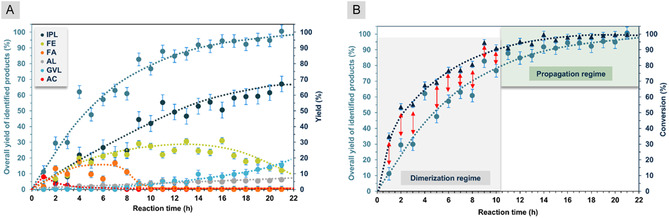
(A) Overall plot of the transfer hydrogenation of furfural to small oxygenates using IPA as reducing agent in the time frame of 21 h, and (B) the comparison of the total yield of all identified products and the conversion plot.

While the screening experiments were conducted over a period of 18 h, we next tested the progress of the reaction using 1 mol% of MSA. From 2 to 7 h the conversion of FF increased from 54% to 77%, respectively (Figure 5A,B). After 9 h, FF was almost fully converted (94%), while after 21 h complete conversion was achieved (Figure 5B). With the longer reaction time, the yield of IPL constantly increased, while FA was only present between 2 and 9 h (Figure 5A,B). We conclude that under acidic conditions FA is mainly converted into FE in an acid‐catalyzed etherification reaction, followed by a slow ring‐opening to yield IPL. After 21 h reaction time, full conversion was detected, with 67% yield of IPL, 15% of GVL, and 12% of FE as main products (Table 2, entry 11). The formation of GVL is reminiscent of a recent study by Nielsen and coworkers in which FF conversion to GVL is achieved using Ru‐MACHO‐BH in EtOH in the presence of H_2_ and H_3_PO_4_ as an acid additive (Figure 2) [22].

To the best of our knowledge our method represents a rare example of the homogenously catalyzed selective TH of FF to IPL as main product under Brønsted acidic conditions. Our method is scalable, when conducted on a 6 mmol scale, 61% of IPL were formed. After purification by simple column chromatography, IPL was isolated in 53% yield (Table 2, entry 12). To clarify the nature of catalysis, we carried out catalyst poisoning experiments to distinguish between homogenous and heterogeneous catalytic pathways. Addition of an excess of Hg(0) or the addition of PPh_3_ (2 mol%), respectively, to the reaction mixture containing 0.5 mol% Ru‐MACHO‐BH, 1 mol% MSA, 2mmol of FF, revealed no changes of the catalytic activity observed previously. In both cases IPL was afforded in 65% yield. This observation conclusively demonstrates a homogenously catalyzed transformation via the entire reaction cascade.

A recent study on the stability of furan derivatives, including FF, in common organic solvents in the presence of acidic (H_2_SO_4_) or basic (NaOH) additives, revealed significant FF degradation in both cases at 80°C [49]. Considering that in our case only 1 mol% of MSA were used, minimal degradation of FF was expected. However, a very interesting phenomena was observed during our measurements to record the overall reaction profile (Figure 5A). In the initial regime of the reaction FE, FA, and the acetal formed from FF and 2 equivalents *i*PrOH (AC), form as intermediates, and are consumed during a given reaction time (Figure 5A). In case of FE, a broad, more equilibrated presence of this intermediate can be seen. In case of FA, a shorter period of formation until 9 h reaction time can be detected. AC as an intermediate is formed rapidly, with low yields and slowly converts further. For GVL a slow increasing formation rate can be observed, which is not surprising, since these oxygenates represents the ‘second half’ of the reaction cascade and are only formed once enough IPL is present. During our mass balance analysis, we concluded that the overall reaction profile shows a classic bending shape, however, the product distribution analysis reveals a nonclosed mass balance considering all forming elements (Figure 5B). In the initial 9 h of the reaction, it is worth noting that the conversion of FF is higher than the overall yields of all identified products (Figure 5A,B). With an average of ∼15%–20% (Figure 5B, red arrows), the conversion curve shows the presence of unidentified products. Because of this insufficiency, we have analyzed with high resolution mass spectrometry, the possible presence of high molecular weight components, most likely intermediately formed dimers and trimers derived from FF, FA, and *i*PrOH (Figures S14 and S15).

**FIGURE 6:**
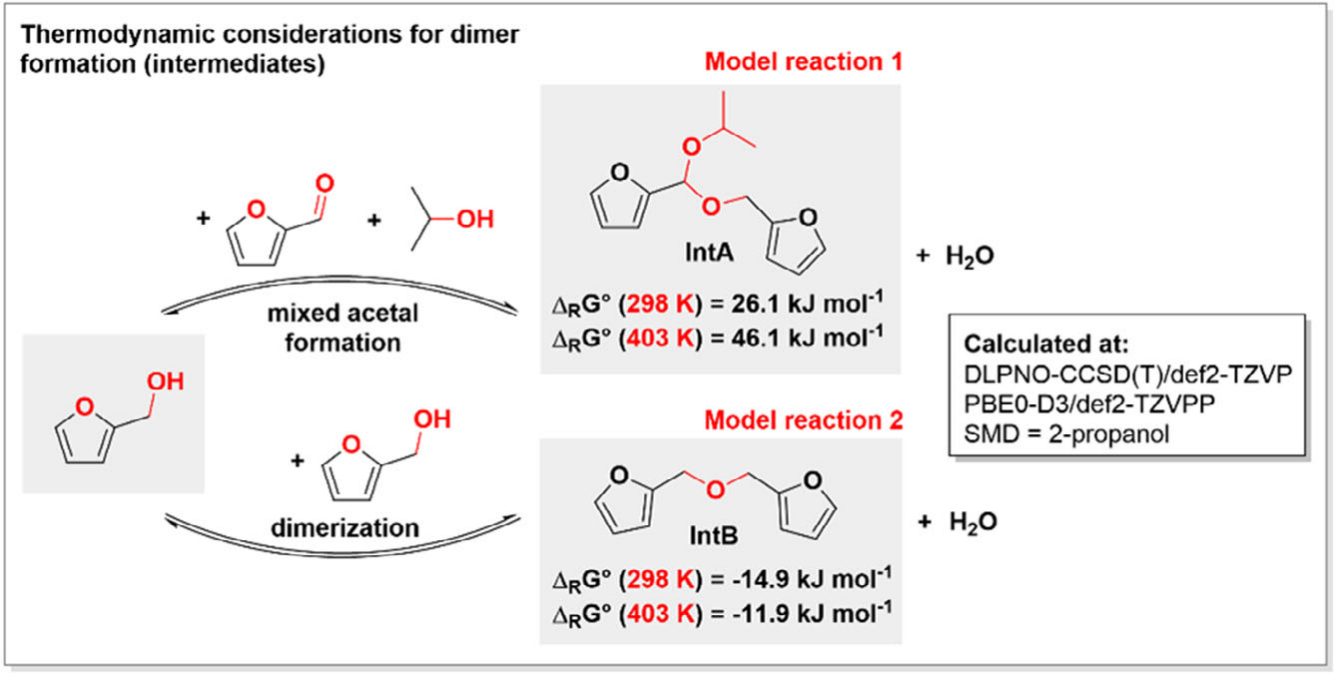
Thermodynamic considerations of the formation of possible intermediates via mixed acetal formation and via dimerization of the intermediately forming partially hydrogenated FA (for detailed description please see ESI, Table S4, Figures S14 and S15).

Based on this investigation we could exclude the presence of oligomer/polymer like materials, and we could clearly identify two main peaks representing the molecular weights of 236.27 and 178.18 g mol^−1^ (Figures S14 and S15). The higher molecular weight component was identified as a mixed acetal, the 2‐((furan‐2‐yl(isopropoxy)methoxy)methyl)furan, which was formed from FF via acetal formation from intermediately formed FA in the presence of IPA as the solvent under acidic conditions (Figure 6, **IntA**). The smaller molecular weight component was recognized as an ether, derived from two molecules of FA, the 2,2′‐(oxybis(methylene))difuran (Figure 6, **IntB**). Indeed, in the initial regime of the reaction profile, the formation of such intermediates (**IntA** and **IntB**) is anticipated, due to the multicomponent nature of the reaction mixture. However, they are consumed during the reaction, since they can re‐enter the catalytic cycle and acting as a possible reservoir for FF and FA as it can be seen in the overall plot of the reaction (Figure 5). Unfortunately, our efforts to directly synthesize or separate **IntA** and **IntB** from the reaction mixtures and characterize them were unsuccessful.

The theoretical analysis (ESI, Table S5) showed that the formation of intermediate **IntA** at lower temperature (298 K) and at the reaction temperature (403 K) is endergonic, indicating the nonspontaneous nature of the reaction and the need of constant external energy input. However, this also shows that the back‐reaction can readily occur, inferring the role of **IntA** as a reservoir for FF and FA. By contrast, the formation of **IntB** is slightly exergonic, showing a thermodynamically more favored reaction to proceed in case of **IntB** in comparison with **IntA** (Figure 6). However, the back reaction from **IntB** to FA is only minimally endergonic, therefore it should proceed readily under the reaction conditions. Both reactions are acid‐catalyzed conversion and the calculated Δ_R_ *G*° values indicate that the formation of such intermediates is predictable under the given reaction conditions. In the propagation regime of the reaction (Figure 5B), the discrepancy between the conversion values and the overall yields are decreasing to minimal or no differences, indicating that the formation of **IntA** and **IntB** is reversible and that the reformed FF and FA are consumed during the reaction. With this interim pattern the reaction is kept in the monomers stage and the formation of high molecular weight products is prevented (humins).

**FIGURE 7:**
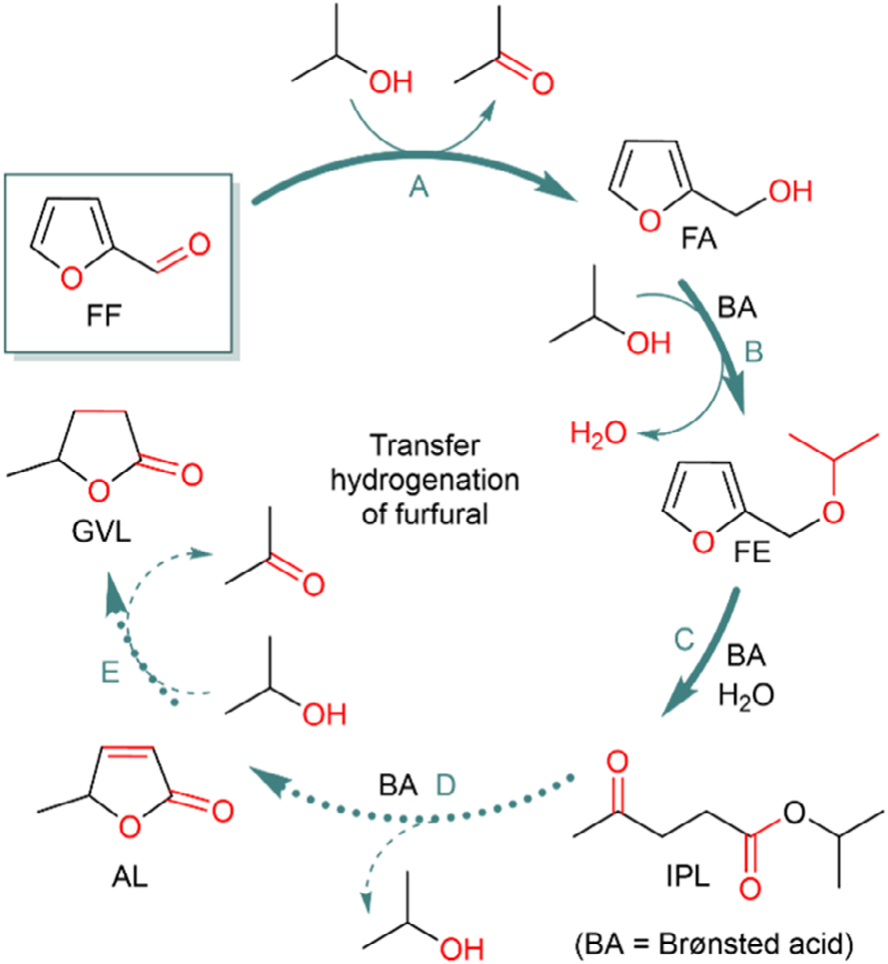
Furfural conversion to small oxygenates via transfer hydrogenation reaction in liquid phase, proposed mechanistic steps.

Based on the recorded reaction profile and the product distribution panel of the reaction cascade within the 21 h reaction time frame, we propose the following reaction mechanism (Figure 7): FA forms from the starting aldehyde (FF) via the TH of the C—O bond (metal catalyzed step) (Figure 7, step **A**) followed by Brønsted acid‐assisted etherification leading to FE (Figure 7, step **B**).

Ring opening and hydrolysis leads to the formation of IPL (Figure 7, step **C**), which goes on in a ring closing reaction catalyzed by a Brønsted acid and produces AL (Figure 7, step **D**). As the end product of the reaction cascade, GVL forms via the metal catalyzed TH of the C—C double bond of AL (Figure 7, step **E**). Other products than those summarized in Figure 2 were not identified, except the intermediately formed species **IntA** and **IntB** (Figure 6), which were analyzed by mass spectroscopy. The reaction cascade was selective in terms of monomers, formation of higher molecular weight components was not observed (Table 2).

Our observations indicated that the conversion of FF to FA is catalyzed by Ru‐MACHO‐BH, while the subsequent transformation of FA to FE, IPL, and AL proceeds in a Brønsted acid‐catalyzed route. The final sub‐step of the reaction cascade, the formation of GVL from AL again necessitates involvement of Ru‐MACHO‐BH (Table 3). To underline this, the conversion of FA to IPL in the absence of Ru‐MACHO‐BH was tested, with respect to different acid additives (Table 3). Using 1 mol% of MSA or H_2_SO_4_, respectively, FA was fully converted to IPL with yields ranging from 80% to 99% (Table 3, entry 4). When increasing the amount of MSA to 1.5 mol%, IPL was formed quantitatively (Table 3, entry 6). When increasing the acid loading further unselective degradation of FA was observed, in line with the recent study on the stability of furan derivatives [49]. We also observed that, acids such as HFIP, H_3_PO_4_, and TFA (Table 3, entry 1–3 and 5) under the same reaction conditions did not lead to complete conversion of FA.

**TABLE 3:** Acid‐catalyzed hydrolysis of furfuryl alcohol in the absence of Ru‐MACHO‐BH at 130°C.

Entry	Acid	Conversion, %	Yield, %		
			FE	IPL	AL
**1**	HFIP	3	–	<1	<1
**2**	H_3_PO_4_	2	–	<1	<1
**3**	TFA	7	–	3	4
**4**	MSA	99	11	80	5
**5**	H_2_SO_4_	99	–	99	1
**6**	MSA^a^	99	<1	95	4

*Note:* Reaction conditions: furfuryl alcohol 1 mmol, 2.5 mL *i*PrOH, 1 mol% acid, 130°C, 16 h.

^a^1.5 mol% MSA. (Product identification/conversion (%)/yield (%) were determined by GC(‐MS) analysis using dodecane as internal standard, for detailed description please see ESI).

Heterogeneous catalysts are significantly dominating the field of catalytic conversion of furfural to small oxygenates [23]. Using heterogeneous systems, the harmonization of the catalytic functions can be positioned in a well‐defined way, and due to the specific adsorption mode and adsorption strength of all reactants, the reaction sequence can proceed in an ordered‐manner. However, in case of a homogeneous catalyst all reactants and products are in the bulk phase, which makes the fine tuning of the reaction conditions very challenging. The newly developed protocol described in here is using FF as the starting material and tries to cover all the catalytic functions which are involved in the reaction cascade under mild conditions, at 130°C, using IPA and only 0.5 mol% of the Ru‐MACHO–BH complex without the usage of external gaseous hydrogen and with minimal formation of humins. Moreover, MSA, the Brønsted acid in the reaction mixture was only used in 1 mol%. In comparison with the closest representatives published already in the literature the current finding represents a greener approach [39, 40]. The Ru‐MACHO–BH complex was used successfully previously for GVL production using molecular hydrogen [28], however under the current reaction conditions it was highly active in IPL formation as well.

## 3 Conclusion

This study demonstrates a homogeneously catalyzed TH method for the stepwise transformation of furfural to isopropyl levulinate, achieving the highest reported selectivity and a yield of 67%. The reaction proceeds under acidic conditions, using only 1 mol% of MSA as Brønsted acid. Upon scaling up the reaction to 6 mmol of furfural, IPL was isolated in 53% yield. Our findings indicate that the Brønsted acid−Ru‐MACHO‐BH catalyst system is highly active for IPL formation. Additionally, we also demonstrated the ester TH of IPL to GVL, achieving 15% yield. Ongoing investigations are focused on elucidating the reaction mechanism further and evaluating the recyclability of the catalytic system to support a more sustainable process.

## Supporting Information

Additional supporting information can be found online in the Supporting Information section. The authors have cited additional references within the Supporting Information [50, 51, 52, 53, 54, 55, 56, 57, 58, 59, 60, 61, 62, 63]. **Supporting Fig. S1:** Transfer hydrogenation of furfural to small oxygenates with Ru‐based complexes. **Supporting Fig. S2:** Calibration curve of FF, GC area ratio of FF to dodecane (as internal standard) vs the amount of FF (in mmol) normalized to the amount of dodecane (in mmol). **Supporting Fig. S3:** Calibration curve of IPL, GC area ratio of IPL to dodecane (as internal standard) vs the amount of IPL (in mmol) normalized to the amount of dodecane (in mmol). **Supporting Fig. S4:** Calibration curve of FE, GC area ratio of IPL to dodecane (as internal standard) vs the amount of FE (in mmol) normalized to the amount of dodecane (in mmol). **Supporting Fig. S5:** Calibration curve of GVL, GC area ratio of IPL to dodecane (as internal standard) vs the amount of GVL (in mmol) normalized to the amount of dodecane (in mmol). **Supporting Fig. S6:** Calibration curve of AL, GC area ratio of IPL to dodecane (as internal standard) vs the amount of AL (in mmol) normalized to the amount of dodecane (in mmol). **Supporting Fig. S7:** Calibration curve of AC, GC area ratio of IPL to dodecane (as internal standard) vs the amount of AC (in mmol) normalized to the amount of dodecane (in mmol). **Supporting Fig. S8:** Calibration curve of FA, GC area ratio of IPL to dodecane (as internal standard) vs the amount of FA (in mmol) normalized to the amount of dodecane (in mmol). **Supporting Fig. S9:** Broadband mass spectra, proving the substantially stronger appearance of the compound according to **IntA** compared to **IntB** compound. (Broadband mass spectrum obtained by direct infusion (+)ESI‐HRMS analysis of the 1:15,000 diluted reaction mixture after 5 h, including a zoom‐in of the mass spectrum is shown for the nominal mass *m*/*z* 201. **Supporting Fig. S10:** Tandem‐Mass spectrum (fragmentation) for structural elucidation of the compound IntA. (Collision‐induced dissociation (CID) spectrum of the precursor ion *m*/*z* 259, collision energy 20.0 eV, isolation width 1 *m*/*z*, obtained by direct infusion (+)ESI‐HRMS analysis of the diluted reaction mixture after 5 h.). **Supporting Fig. S11:** ^1^H NMR spectrum for IPL (CDCl_3_, 300 MHz, r.t.). **Supporting Fig. S12:** ^1^H NMR spectrum of AC (CDCl_3_, 300 MHz, r.t.). **Supporting Fig. S13:** ^13^C NMR spectrum of AC (CDCl_3_, 75.5 MHz, r.t.). **Supporting Fig. S14:** Representative GC spectra of the product distribution after 3 h reaction time without any additional purification before analysis. The corresponding retention times: 1.7 min/solvent, 4.6 min/FF, 5.2 min/FA, 7.3 min/AL, 7.6 min/FE, 10.7 min/IPL, 11.6 min/AC, 12.3 min/dodecane (internal standard), 16.3 min/IntB, 18.4/IntA. **Supporting Fig. S15:** Representative GC spectra of the product distribution after 21 h reaction time without any additional purification before analysis. The corresponding retention times: 1.7 min/solvent, 4.4 min/FF, 7.4 min/AL, 7.6 min/FE, 7.7 min/GVL, 10.8 min/IPL, 12.3 min/dodecane (internal standard). **Supporting Table S1:** Summary of all chemicals used in the current study. **Supporting Table S2:** Screening of dichloride complexes for the transfer hydrogenation reaction. **Supporting Table S3:** Exact values of the overall plot of the transfer hydrogenation of furfural. **Supporting Table S4:** Transfer hydrogenation of furfural at higher FF concentrations and evaluation of the volume of the reaction vessel. **Supporting Table S5:** Summary of calculated data, including electronic energies and thermal corrections.

## Funding

This study was supported by Horizon 2020 Framework Programme (101119277).

## Conflicts of Interest

The authors declare no conflicts of interest.

## Supporting information

Supplementary Material

## Data Availability

The data that support the findings of this study are available in the supplementary material of this article.
