# Supplementary material for: Selective Transfer Hydrogenation of Furfural to Isopropyl Levulinate: An In Situ One‐Pot Cascade Approach
Source: ChemSusChem. 2026 Mar 1;19(5):e202502378. doi: 10.1002/cssc.202502378 (PMC12950357; doi:10.1002/cssc.202502378)
Supplement: Supplementary file 1 — Supplementary Material [file CSSC-19-e202502378-s001.pdf]

Supporting Information

Selective transfer hydrogenation of furfural to isopropyl levulinate: An *in situ* one-pot cascade approach

Saravanan Subramaniyan,[a] Christian Hering-Junghans,[a,]* and Eszter Baráth[a,]*

[a] Leibniz Institut für Katalyse e.V. (LIKAT) Albert Einstein Str.29a, D-18059 Rostock (Germany)

E-mail: Christian.Hering-Junghans@catalysis.de; Eszter.Barath@catalysis.de

Table of content

**Analytical and experimental methods**	**S3**
**Mode of calculation**	**S4**
**Materials**	**S5**
**Experimental details**	**S6**
**Quantitative analysis of all identified monomers**	**S8**
**Representative HR-MS spectra**	**S11**
**Scale-up experiment of FF to IPL**	**S12**
**Synthesis of AC**	**S13**
**Representative GC spectra**	**S15**
**Computational details**	**S16**
**General remarks**	**S17**
**References**	**S18**

Analytical and experimental methods

GC

The GC samples was prepared by adding 50 µL dodecane (normal dodecane) to the reaction mixture, which was then stirred for a minute and the excess of acid quenched by adding water and then the organic phase (1 mL) was taken to GC analysis. The quantitative and qualitative analysis was made with an Agilent 7890B gas chromatograph equipped with a flame ionization detector (FID) and with an Agilent 5977A mass spectrometer; 1 µL of the liquid sample was injected into a HP-5 normal phase silica column (30 m × 320 µm × 0.25 µm) at an inlet temperature of 280°C, using a split ratio 50 (He). The corresponding heating program was the following: step/heating rate (°C min^{-1})/ temperature (°C)/ hold time (min): 1/-/50/5 – 2/10/250/-.

NMR

NMR spectra were recorded on a Bruker AVANCE 300 MHz or 400 MHz instrument and were referenced internally to the deuterated solvent (^{13}C ($CDCl_3$) δ_{ref} = 77.1 ppm) (^{1}H NMR ($CDCl_3$) δ_{ref} = 7.26 ppm). All measurements were carried out at room temperature unless denoted otherwise NMR signals were assigned using experimental data (e.g. chemical shifts, coupling constants, integrals where applicable).

HR-MS

HR-MS analysis was performed on a timsTOF flex instrument from Bruker Daltonics equipped with an Apollo II ESI source (Bruker Daltonics, Bremen, Germany). Samples were diluted 1:15,000 (v/v) in methanol. Mass spectrometric data were recorded in ESI positive from *m/z* 50−800 with a direct-infusion ion source setup. The ionization parameters were set as follows: capillary voltage −3500 V, nebulizer gas pressure 0.6 bar, drying gas flow rate 3.5 L min^{-1} and drying gas temperature 200 °C. Data were acquired for 10 minutes per measurement with a rate value of 0.5 Hz, resulting in averaged mass spectra from ~300 scans. The resolving power of the timsTOF flex instrument is ~40,000 at *m/z* 1222 and the mass accuracy < 2 ppm with external calibration. For collision-induced dissociation (CID), ions of a 1 Da mass window were isolated by the quadrupole and fragmented inside the CID cell with collision energy of 20 eV.

Experimental method

For a typical transfer hydrogenation reaction experiment, in a glove box, Ru-MACHO-BH (0.005 eq, 5.86 mg, 0.5 mol%) was transferred into a pressure tube (25 mL) equipped with a magnetic stir bar and the tube was sealed with an-air-tight screw-cap. Afterwards dry *i*PrOH (5 mL) was added on a Schlenk-line. Next furfural (2 mmol, 0.165 mL) and methanesulfonic acid (0.004 M, 1.3 µL, 1mol%) were added to the reaction mixture, giving a yellow to brown suspension. Afterwards the pressure tube was sealed and was placed in an aluminium heating block for a certain time (please see experimental section). After completion, the pressure tube was cooled to ambient temperature and the pressure was carefully released, dodecane (50 µL) was added as an internal standard, next the reaction was quenched with water (1 mL). The sample was dried over Na_2SO_4 for GC-analysis an aliquot of 1 mL was taken.

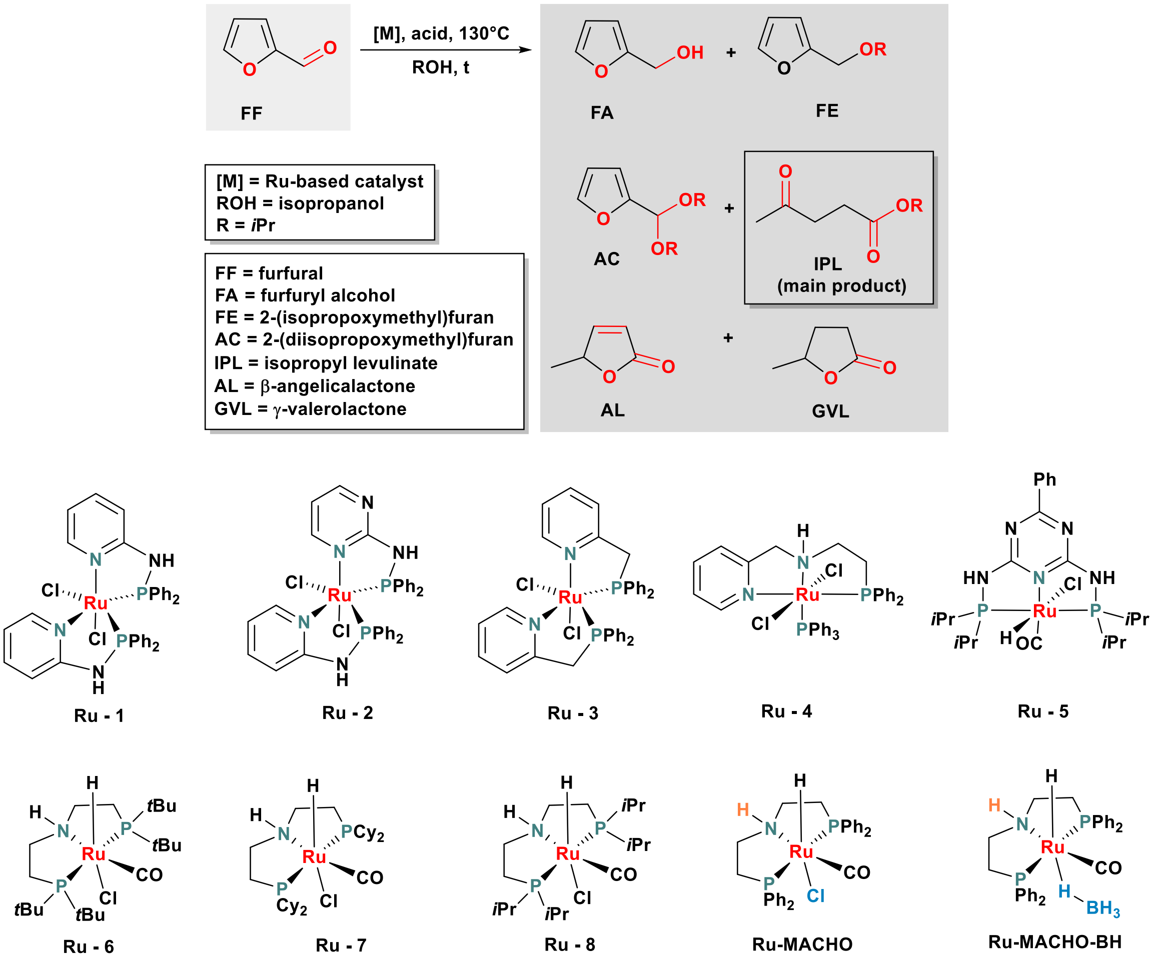

Fig. S1 Transfer hydrogenation of furfural to small oxygenates with Ru-based complexes.

Mode of calculation

$$\textbf{yield} = \frac{mmol\ of\ product}{mmol\ of\ furfural}\ x\ 100\ (\%)$$

$$\textbf{mmol of product} = \frac{area\ of\ product}{area\ of\ standard}\ x\ \frac{mmol\ of\ standard}{RF\ value\ of\ product}$$

$$\textbf{conversion} = \frac{mmol\ of\ initial\ starting\ material - \ mmol\ of\ starting\ material\ left}{mmol\ of\ initial\ starting\ material}\ x\ 100\ (\%)$$

Materials

Table S1. Summary of all chemicals used in the current study.

Substance	Origin	Purity
*i*PrOH	Thermo Scientific, extra dry, 99.5%	Used as received
n-pentane, *n*-hexane	local trade	Used as received
Ru-1[30]		Synthesized
Ru-2[30]		Synthesized
Ru-3[30]		Synthesized
Ru-4	Sigma Aldrich	Used as received
Ru-5[32a]		Synthesized
Ru-6	TCI	Used as received
Ru-7	TCI	Used as received
Ru-8	TCI	Used as received
Ru-MACHO	TCI	Used as received
Ru-MACHO-BH	TCI	Used as received
methanesulfonic acid	Sigma Aldrich, 99%	Used as received
furfural	Sigma Aldrich, ACS reagent, 99%	Distilled
p-toluenesulfonic acid monohydrate	abcr, 97%	Used as received
formic acid	Sigma Aldrich (reagent grade)	Used as received
phosphoric acid	abcr,	Used as received
trifluoroacetic acid	Sigma Aldrich	Used as received
hexafluoroisopropanol	Flurochem	Used as received
trifluoroethanol	chemPUR 99%	Used as received
Hg	Carl ROTH GmbH	Used as received
PPh_3	Sigma Aldrich, 97%	Used as received
$RuCl_2(PPh_3)_3$	BLDpharm, 97%	Used as received
furfuryl alcohol	Sigma Aldrich, 98%	Used as received
levulinic acid	Sigma Aldrich, 98%	Used as received
β-angelica lactone	BLDpharm	Used as received

Experimental details

Table S2. Screening of dichloride complexes for the transfer hydrogenation reaction.

Entry	Catalyst	Conversion (%)	Yield of FA (%)	Yield of AC (%)	Humins (%)
1	Ru-1	54	1	32	21
2	Ru-2	53	1	33	19
3	Ru-3	48	-	30	18
4	Ru-4	32	-	32	-

Reaction condition: furfural (13.7 mmol), Ru (1 mol%), NaOH (0.274 mmol), *i*PrOH (8 mL), 100 °C, 10 hours.

Table S3. Exact values of the overall plot of the transfer hydrogenation of furfural.

Reaction time (h)	Conversion (%)	Yield (%)					
		FA	AC	FE	IPL	AL	GVL
1	35 (±3)	2 (±2)	8 (±2)	< 1	< 1	-	-
2	53 (±2)	13 (±2)	4 (±2)	7 (±4)	6 (±3)	< 1	-
3	55 (±3)	10 (±4)	3 (±2)	9 (±3)	8 (±3)	< 1	-
4	67 (±4)	14 (±3)	< 1	7 (±3)	18 (±3)	2 (±1)	-
5	69 (±2)	17 (±2)	< 1	17 (±3)	19 (±3)	2 (±1)	-
6	77 (±1)	7 (±2)	< 1	21 (±3)	27 (±3)	2 (±1)	-
7	77 (±2)	17 (±2)	-	22 (±3)	22 (±3)	2 (±1)	-
8	80 (±2)	11 (±2)	-	22 (±3)	25 (±3)	2 (±1)	< 1
9	94 (±2)	< 1	-	29 (±3)	44 (±3)	4 (±1)	4 (±2)
10	91 (±3)	< 1	-	27 (±3)	42 (±4)	4 (±1)	3 (±2)
11	91 (±2)	< 1	-	22 (±3)	55 (±3)	5 (±1)	5 (±2)
12	95 (±2)	< 1	-	25 (±3)	49 (±5)	5 (±1)	5 (±2)
13	93 (±2)	-	-	30 (±3)	47 (±4)	4 (±1)	4 (±2)
14	98 (±1)	-	-	24 (±3)	53 (±4)	5 (±1)	10 (±2)
15	96 (±2)	-	-	24 (±3)	55 (±4)	5 (±1)	6 (±3)
16	97 (±2)	-	-	31 (±3)	51 (±4)	5 (±1)	8 (±3)
17	98 (±2)	-	-	21 (±3)	58 (±4)	5 (±1)	10 (±2)
18	98 (±2)	-	-	18 (±3)	59 (±4)	5 (±1)	10 (±2)
19	99 (±2)	-	-	18 (±3)	59 (±4)	5 (±1)	10 (±2)
20	99 (±2)	-	-	14 (±3)	60 (±3)	5 (±1)	14 (±2)
21	99 (±1)	-	-	12 (±3)	67 (±2)	6 (±1)	15 (±2)

Reaction condition: furfural (2mmol), Ru-MACHO-BH (0.5 mol%), methanesulfonic acid (0.004M/1mol%), *i*PrOH (5mL), 130 °C for the reaction stirred for the period indicated. Yield and contribution determined by GC-FID using dodecane as internal standard and all the samples analysed by GC(-MS). **Note**: each reaction was performed five times, the average error bars are reported in parentheses.

Table S4. Transfer hydrogenation of furfural at higher FF concentrations and evaluation of the volume of the reaction vessel.

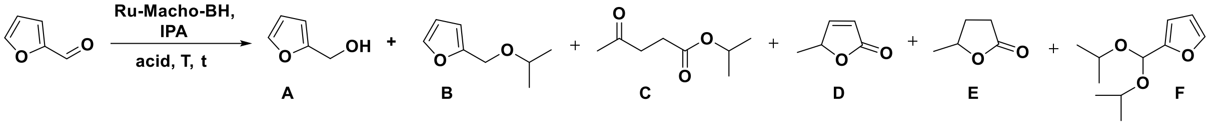

Entry	Reaction time (h)	T (°C)	Conversion (%)	GC Yield (%)					
				A	B	C	D	E	F
1 [a]	5	130	59	11	13	9	0	0	17
2 [a]	12	130	70	1	16	24	1	1	12
3 [a]	21	130	85	1	14	40	2	3	5
4 [b]	21	100	78	59	13	0	0	0	6
5 [c]	5	130	43	0	7	6	0	0	27
6 [c]	12	130	72	0	14	29	1	0	9
7 [c]	21	130	78	0	18	31	2	0	6
8 [d]	21	100	30	0	3	2	0	0	24
9 [e]	5	130	71	16	27	0	2	0	11
10 [e]	12	130	100	43	37	3	0	0	0
11 [e]	21	130	100	0	9	59	4	11	0
12 [f]	21	100	85	54	16	5	0	0	4
13 [g] Reference experiment	21	130	100	0	12	67	6	15	0

Corresponding reaction conditions:

a: furfural 2 mmol, 3 mL IPA, 0.5 mol% cat., 0.02 mmol (MSA), 25 mL pressure tube.

b: furfural 2 mmol, 3 mL IPA, 0.5 mol% cat., 0.02 mmol (MSA), 25 mL pressure tube.

c: furfural 2 mmol, 3 mL IPA, 0.5 mol% cat., 0.02 mmol (MSA), 50 mL pressure tube.

d: furfural 2 mmol, 3 mL IPA, 0.5 mol% cat., 0.02 mmol (MSA), 50 mL pressure tube.

e: furfural 2 mmol, 3 mL IPA, 0.5 mol% cat., 0.02 mmol (MSA), 10 mL pressure tube.

f: furfural 2 mmol, 3 mL IPA, 0.5 mol% cat., 0.02 mmol (MSA), 10 mL pressure tube.

g: furfural 2 mmol, 5 mL IPA, 0.5 mol% cat., 0.02 mmol (MSA), 130 °C, 25 mL pressure tube.

Quantitative analysis of all identified monomers

In a vial a given mmol of a small oxygenate (such as: FF, IPL, FE, GVL, AL, AC, FA, **Fig. S2** - **Fig.S8**) was taken, and then 5mL of *i*PrOH and 50 µL of dodecane (used as an internal standard) were added. After shaking the mixture, an aliquot (1mL) was transferred to a GC vial and analysed. The corresponding GC-area of a given compound and dodecane was used to calculate the corresponding amount (mmol) of the compound, which was then used to determine the actual concentration. (The same procedure was used for all the other monomers.)

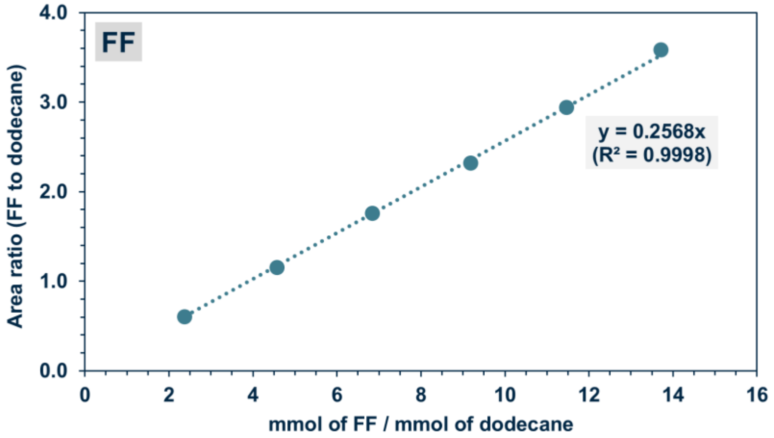

Fig. S2 Calibration curve of FF, GC area ratio of FF to dodecane (as internal standard) vs the amount of FF (in mmol) normalized to the amount of dodecane (in mmol).

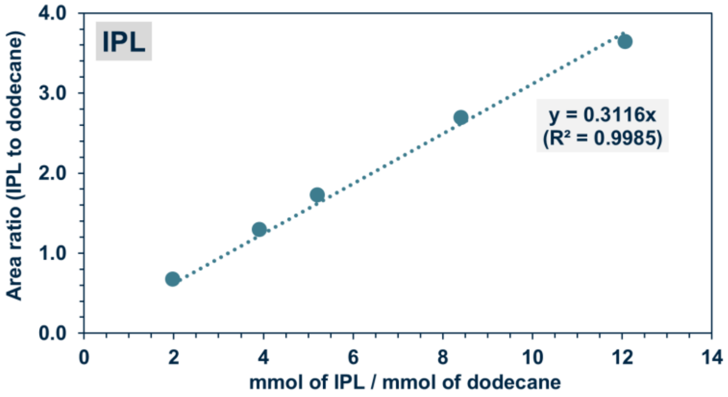

Fig. S3 Calibration curve of IPL, GC area ratio of IPL to dodecane (as internal standard) vs the amount of IPL (in mmol) normalized to the amount of dodecane (in mmol).

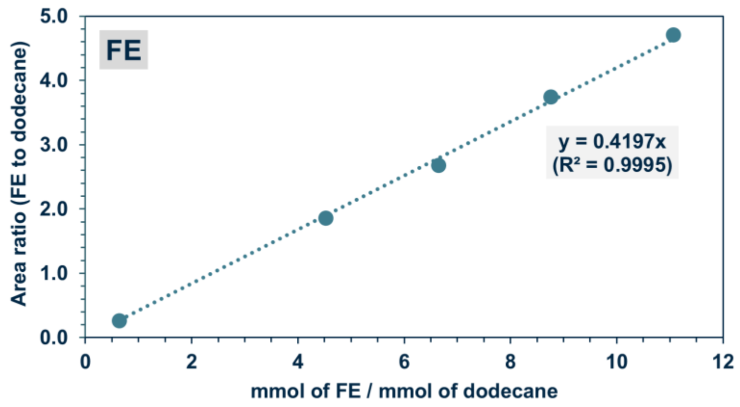

Fig. S4 Calibration curve of FE, GC area ratio of IPL to dodecane (as internal standard) vs the amount of FE (in mmol) normalized to the amount of dodecane (in mmol).

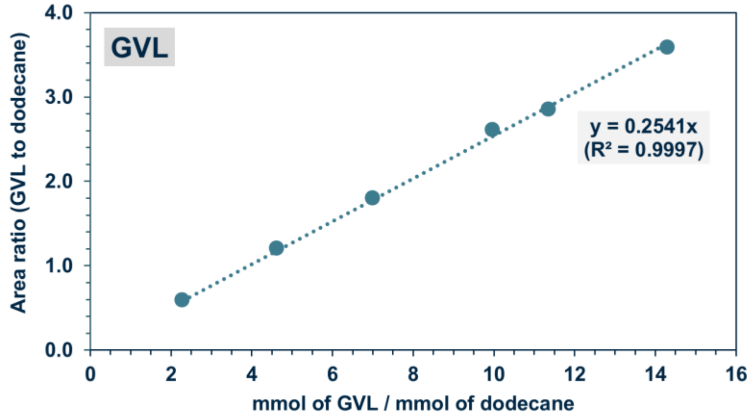

Fig. S5 Calibration curve of GVL, GC area ratio of IPL to dodecane (as internal standard) vs the amount of GVL (in mmol) normalized to the amount of dodecane (in mmol).

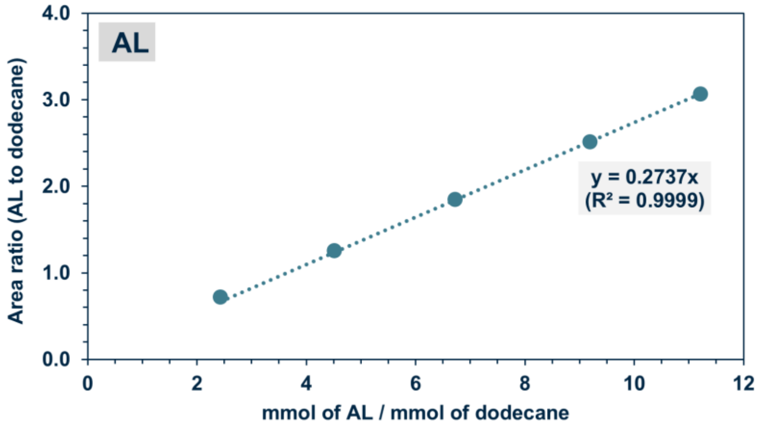

Fig. S6 Calibration curve of AL, GC area ratio of IPL to dodecane (as internal standard) vs the amount of AL (in mmol) normalized to the amount of dodecane (in mmol).

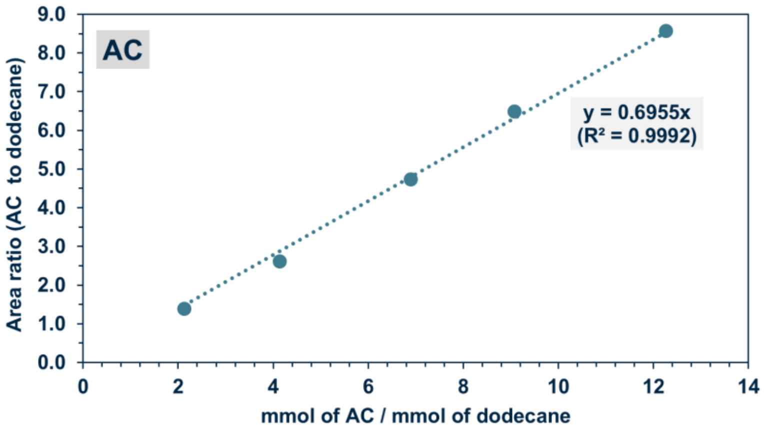

Fig. S7 Calibration curve of AC, GC area ratio of IPL to dodecane (as internal standard) vs the amount of AC (in mmol) normalized to the amount of dodecane (in mmol).

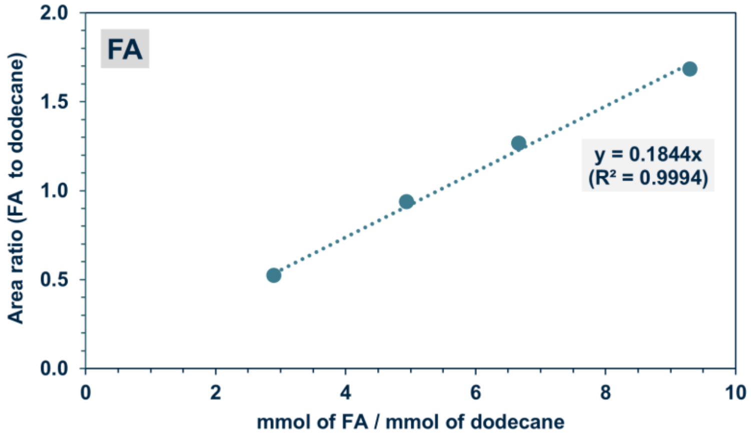

Fig. S8 Calibration curve of FA, GC area ratio of IPL to dodecane (as internal standard) vs the amount of FA (in mmol) normalized to the amount of dodecane (in mmol).

Representative HR-MS spectra

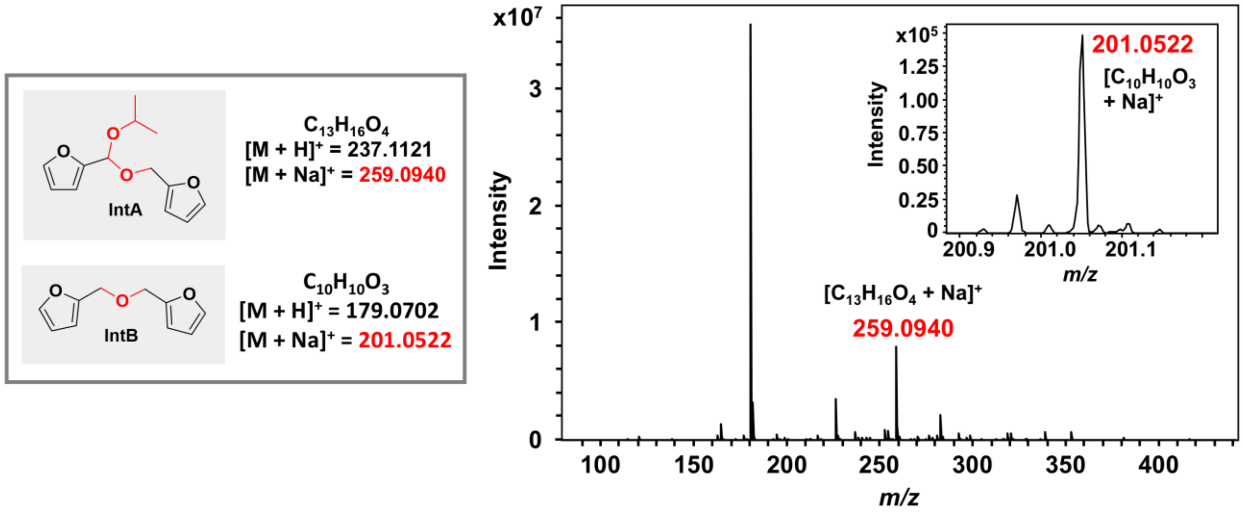

Fig. S9 Broadband mass spectra, proving the substantially stronger appearance of the compound according to **IntA** compared to **IntB** compound. (Broadband mass spectrum obtained by direct infusion (+)ESI-HRMS analysis of the 1:15,000 diluted reaction mixture after 5 h, including a zoom-in of the mass spectrum is shown for the nominal mass *m/z* 201.)

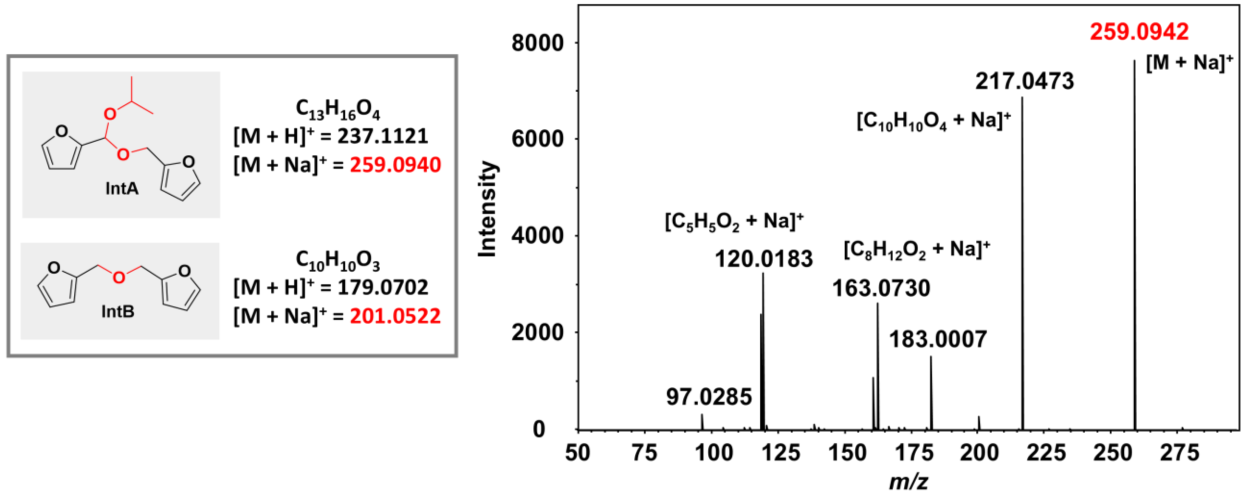

Fig. S10 Tandem-Mass spectrum (fragmentation) for structural elucidation of the compound **IntA**. (Collision-induced dissociation (CID) spectrum of the precursor ion *m/z* 259, collision energy 20.0 eV, isolation width 1 *m/z*, obtained by direct infusion (+)ESI-HRMS analysis of the diluted reaction mixture after 5 h.)

Scale-up experiment of FF to IPL

In a glove box, Ru-MACHO-BH (17.6 mg, 0.5 mol%) was transferred into a pressure tube (50 mL) equipped with a magnetic stir bar and the tube was sealed with an-air-tight screw-cap. Afterwards dry *i*PrOH (15 mL) was added on a Schlenk line. Next furfural (6 mmol, 0.497 mL) and methanesulfonic acid (7.8 µL, 2 mol%) were added to the reaction mixture, giving a yellow to brown suspension. Afterwards the pressure tube was sealed and was placed in an oil bath (130 °C) for 21 hours. Afterwards the pressure tube was cooled to ambient temperature and the pressure was carefully released. Next the reaction was quenched with distilled water. The aqueous phase was removed from the organic phase and dried over Na_2SO_4. After the volatiles were removed in vacuo, the crude product was purified by column chromatography on silica using an ethyl acetate : *n*-hexane (3:97) eluent. The yielded IPL was a colorless oily material. Isolated yield: 0.50 g (3.18 mmol, 53 %).

^{1}H NMR ($CDCl_3$, 300.1 MHz): δ 5.23 (m, 1H), 2.49 (m, 2H), 2.32 (m, 2H), 1.75 (s, 3H), 1.13 (d, *J* = 6.3 Hz, 6H).**^{13}C{^{1}H} NMR** ($CDCl_3$, 75.5 MHz): δ 204.4 (s), 171.5 (s), 67.2 (s), 37.3 (s), 28.8 (s), 28.1 (s), 21.4 (s).

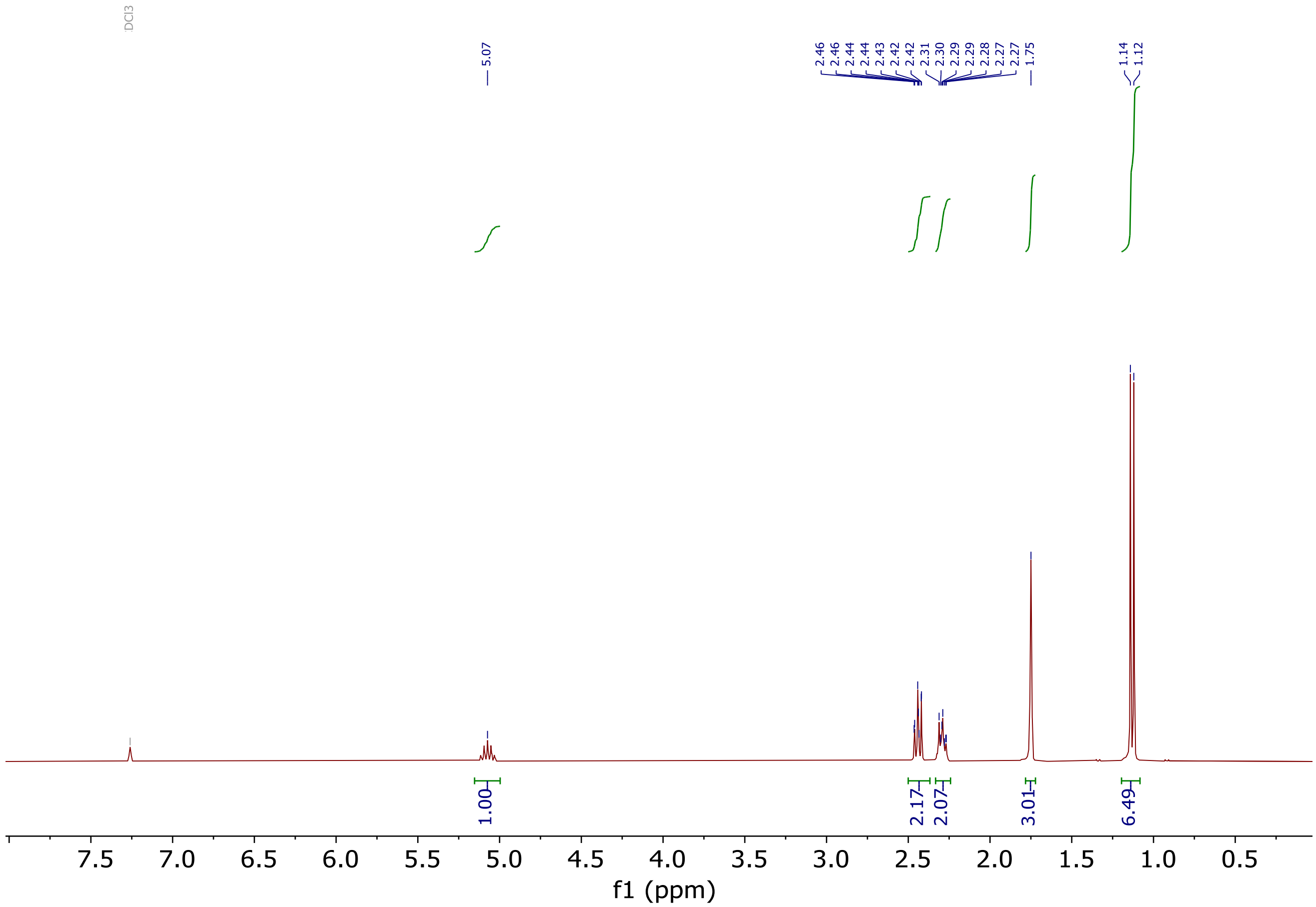

Fig. S11 ^{1}H NMR spectrum for IPL ($CDCl_3$, 300 MHz, r.t.)

Synthesis of AC

To furfural (4.34 mL, 52.5 mmol) in a Schlenk flask equipped with a magnetic stir bar, *i*PrOH (10 mL, 131 mmol) and *n*-hexane (70 mL) were added at room temperature. Next, molecular sieves (10.3 mg, 5 Å) and *p*-toluenesulfonic acid (1.013 g, 5.3 mmol) were added, and the reaction mixture was stirred at room temperature for 2.5 hours. Afterwards the volatiles were removed under reduced pressure, the crude product was separated by flash column chromatography using ethyl acetate : *n*-hexane (3:97) as an eluent. Pure AC was obtained after Kugelrohr distillation (55 °C, 5.60 x 10^{-4} mbar) as a colourless oily material. Isolated yield: 7.01 g (35.31 mmol, 67 %). The NMR data is in line with previous reports.[50] Without further purification furfural diisopropyl diacetal was used as a standard for GC-calibration.

^{1}H NMR ($CDCl_3$, 300.1 MHz): *δ* = δ 7.39 (dd, *J* = 1.8, 0.9 Hz, 1H), 6.41 (m, 1H), 6.34 (dd, *J* = 3.3, 1.8 Hz, 1H), 5.59 (d, *J* = 0.7 Hz, 1H), 3.95 (s, 2H), 1.17 (t, *J* = 6.0 Hz, 13H). **^{13}C{^{1}H} NMR** ($CDCl_3$, 75.5 MHz): *δ* = δ 153.0 (s), 142.1 (s), 110.0 (s), 107.5 (s), 93.8 (s), 68.0 (s), 23.0 (s), 22.4 (s).

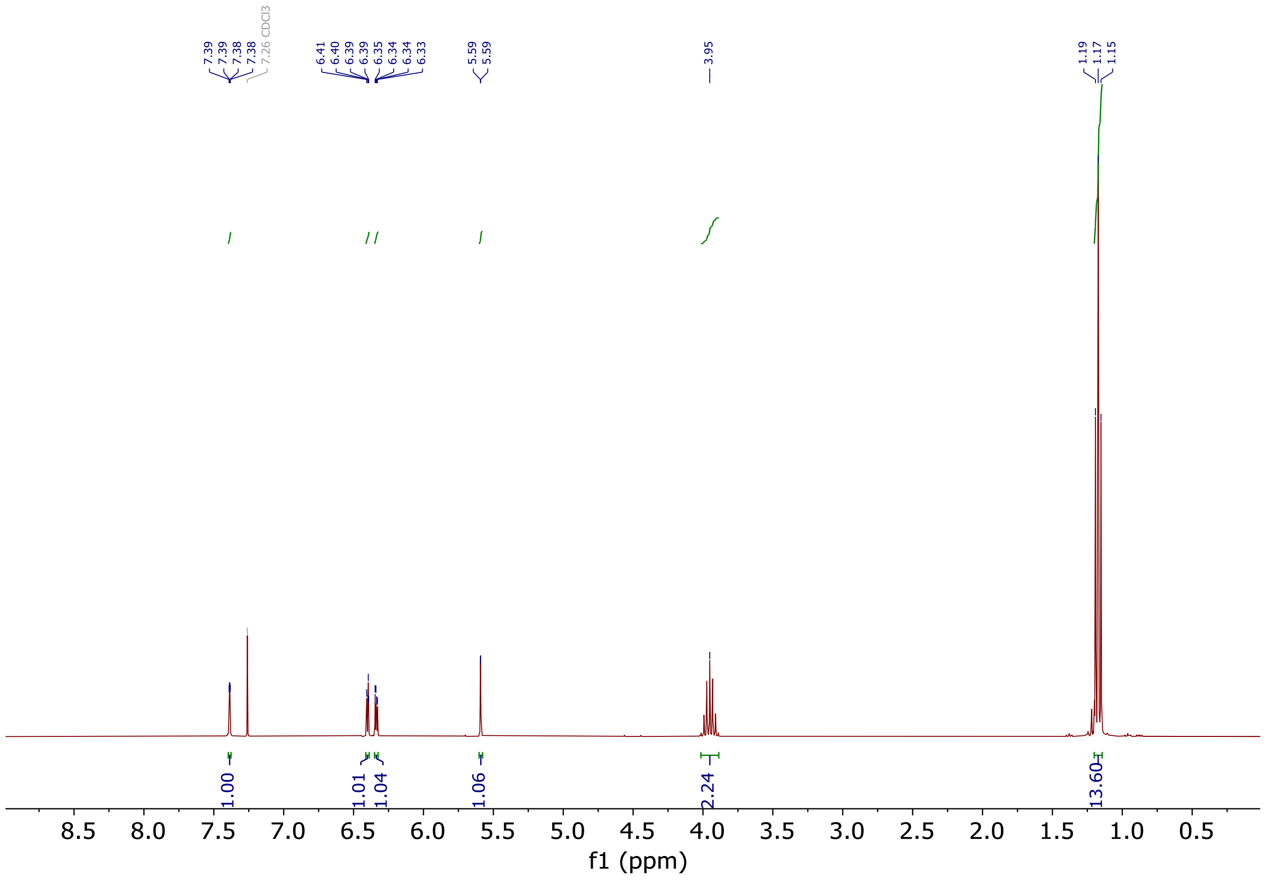

Fig. S12 ^{1}H NMR spectrum of AC ($CDCl_3$, 300 MHz, r.t.).

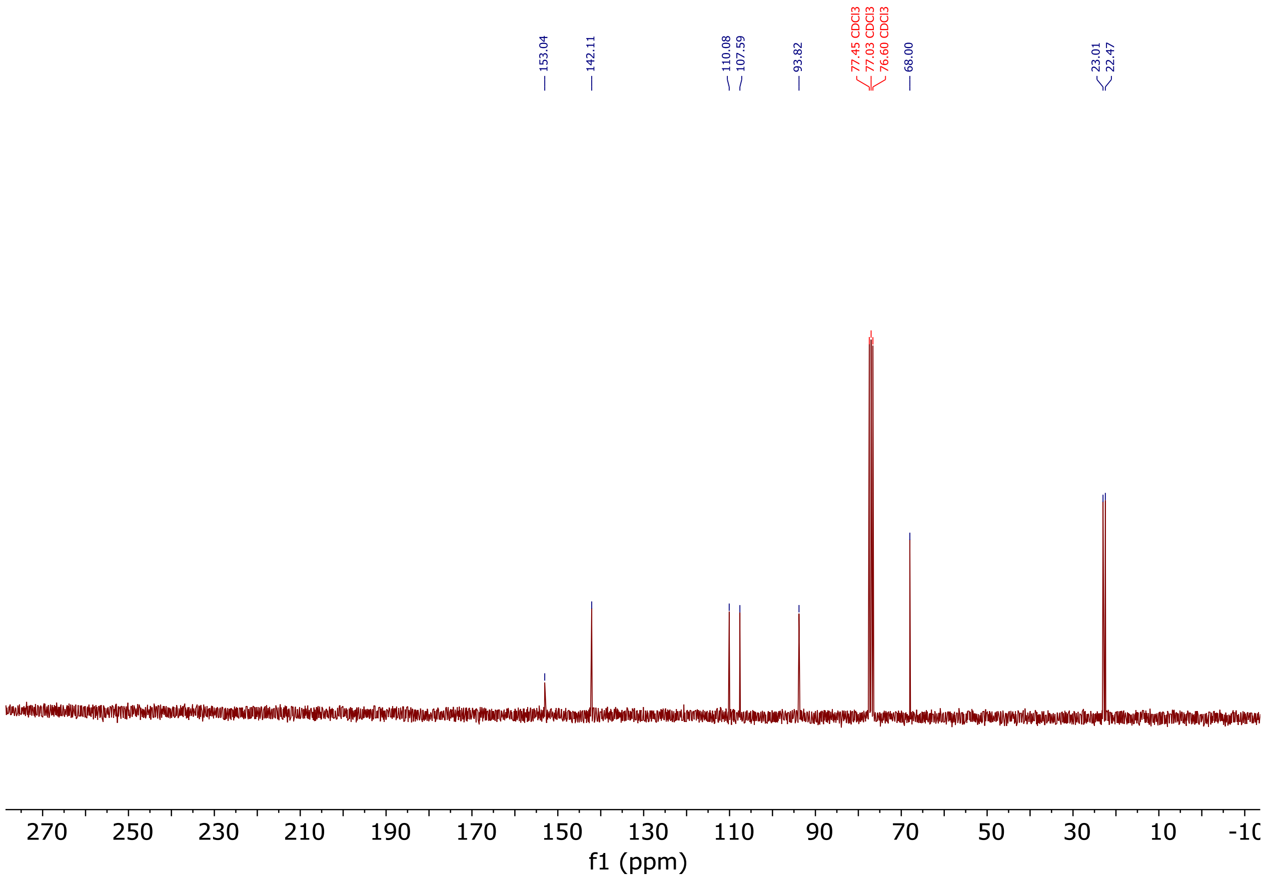

Fig. S13 ^{13}C NMR spectrum of AC ($CDCl_3$, 75.5 MHz, r.t.).

Representative GC spectra

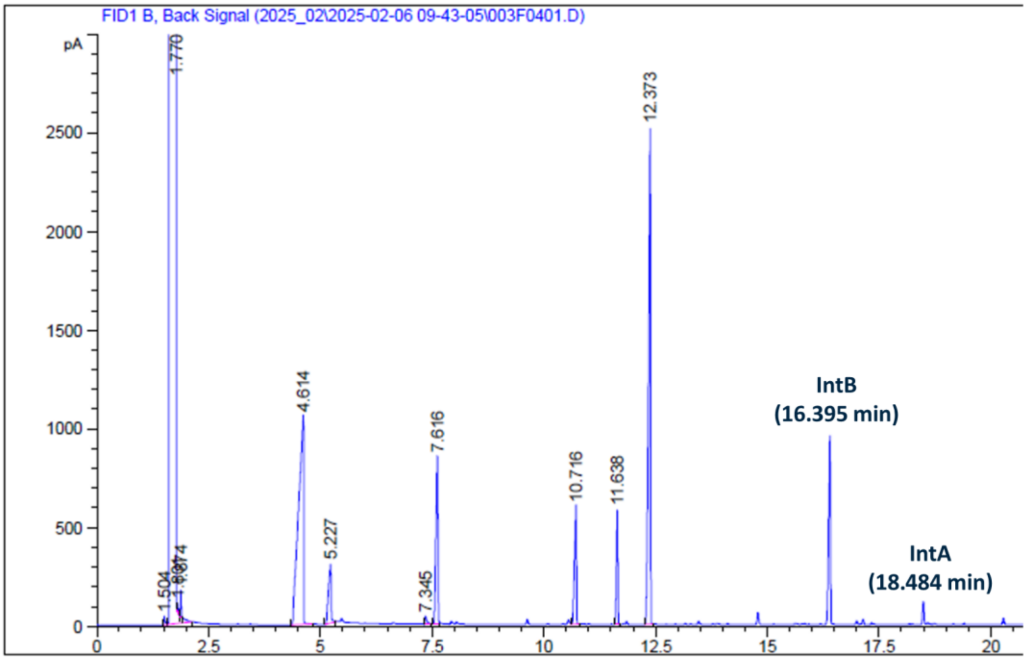


```
=====================================================================
                          Area Percent Report
=====================================================================

Sorted By              :      Signal
Multiplier             :      1.0000
Dilution               :      1.0000
Do not use Multiplier & Dilution Factor with ISTDs


Signal 1: FID1 B, Back Signal
```

Peak #	RetTime [min]	Type	Width [min]	Area [pA*s]	Height [pA]	Area %
1	1.504	BV	0.0267	84.38714	43.33207	0.01446
2	1.770	VB S	0.0908	5.59843e5	7.61091e4	95.95907
3	1.801	BV X	0.0216	52.90068	40.72687	0.00907
4	1.874	VB X	0.0187	154.59073	131.38370	0.02650
5	4.614	BB	0.0960	8238.03223	1056.38721	1.41203
6	5.227	BB	0.0598	1274.18994	298.79788	0.21840
7	7.345	BB	0.0458	129.90178	41.16643	0.02227
8	7.616	BV	0.0464	2648.90112	848.17957	0.45403
9	10.716	VB	0.0443	1806.93066	596.97070	0.30971

Fig. S14. Representative GC spectra of the product distribution after 3h reaction time without any additional purification before analysis. The corresponding retention times: 1.7min/solvent, 4.6min/FF, 5.2min/FA, 7.3min/AL, 7.6min/FE, 10.7min/IPL, 11.6min/AC, 12.3min/dodecane (internal standard), 16.3min/IntB, 18.4/IntA.

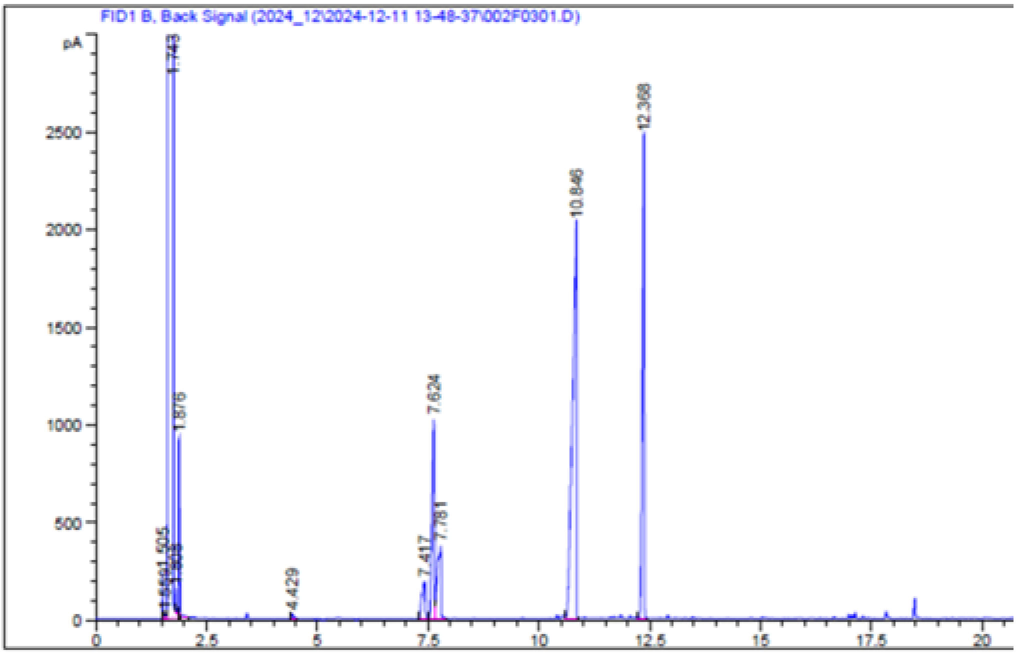


```
=====================================================================
                         Area Percent Report
=====================================================================

Sorted By             :      Signal
Multiplier            :      1.0000
Dilution              :      1.0000
Do not use Multiplier & Dilution Factor with ISTDs


Signal 1: FID1 B, Back Signal
```

Peak #	RetTime [min]	Type	Width [min]	Area [pA*s]	Height [pA]	Area %
1	1.505	BV	0.0123	185.73178	233.69696	0.04038
2	1.559	VV	0.0428	71.89613	22.81606	0.01563
3	1.743	VB S	0.0980	4.31337e5	7.33531e4	93.78423
4	1.808	BV X	0.0103	72.66039	115.69234	0.01580
5	1.876	VB X	0.0207	1218.18213	904.58417	0.26487
6	4.429	BB	0.0495	82.79256	24.47363	0.01800
7	7.417	BV	0.0695	1020.04584	193.59363	0.22179
8	7.624	VV	0.0535	3537.52588	1019.80267	0.76915
9	7.781	VB	0.0742	2173.04248	371.81531	0.47248

Fig. S15. Representative GC spectra of the product distribution after 21h reaction time without any additional purification before analysis. The corresponding retention times: 1.7min/solvent, 4.4min/FF, 7.4min/AL, 7.6min/FE, 7.7min/GVL, 10.8min/IPL, 12.3min/dodecane (internal standard).

Computational details
General remarks

Computations were carried out using Gaussian16[51] and ORCA 4.2.1.[52]

Structure optimizations employed the hybrid DFT functional PBE0[53-55] in conjunction with Grimme's dispersion correction D3(BJ)[56,57] and the def2-TZVPP basis set[58] (notation PBE0-D3/def2-TZVPP). All structures were fully optimized and confirmed as minima by frequency analyses.

More accurate estimates of the electronic energy were obtained by single-point DLPNO-CCSD(T)/def2-TZVP[59-61] computations (notation DLPNO-CCSD(T)/def2-TZVP//PBE0-D3/def2-TZVPP). The $T1$ diagnostic was evaluated in each case to ensure reliable results (empirically, CCSD(T) results with $T1$ values smaller than 0.02 are considered reliable).[62] Solvent corrections were calculated at the PBE0-D3/def2-TZVPP level of theory using the SMD model for 2-propanol ($\Delta G_{solv} = E_{tot,solv} - E_{tot,gas}$)[63] Thermal corrections to the Gibbs free energy at T = 403 K were obtained at the PBE0-D3/def2-TZVPP level of theory. The Gibbs free energy was calculated as follows: $E_{CCSD(T)} + \Delta G_{solv} + \Delta G_T$.

Please note that all computations were carried out for single, isolated molecules in the gas phase (ideal gas approximation). There may well be significant differences between gas phase and condensed phase.

Table S5. Summary of calculated data, including electronic energies and thermal corrections.

Compound	*PG*	E_{tot}[a]	ΔG_{298}[b]	ΔG_{403}[b]	$E_{CCSD(T)}$[a]	ΔG_{solv}
**FF**	C_1	-343.1050	0.0507	0.0375	-342.7858	-0.0105
**FA**	C_1	-344.3094	0.0720	0.0576	-343.9845	-0.0117
**2-propanol**	C_1	-194.2143	0.0807	0.06825	-0.0097	-0.0097
**H_2O**	C_{2v}	-76.3811	0.0040	-0.0038	-76.3267	-0.0113
**IntA**	C_1	-805.2662	0.2240	0.1993	-804.4831	-0.0164
**IntB**	C_1	-612.2451	0.1425	0.1227	-611.6499	-0.0126

[a] Total SCF energy in a.u. [b] Thermal correction to Gibbs energy in a.u. (298 K unless stated otherwise).

The formation of mixed acetal **IntA** (derived from FF, FA and 2-propanol) and **IntB** (from 2 molecules of FA) was considered at 298 K and 403 K. The Gibbs free energy was considered using a solvation model for 2-propanol at the DLPNO-CCSD(T)/def2-TZVP//PBE-D3/def2-TZVPP(2-propanol) level of theory.

References

50 M. Harmata, D. E. Jones, *J. Org. Chem* 1997, **62**, 1578.

51 *Gaussian 09, Revision E.01*, M. J. Frisch, G. W. Trucks, H. B. Schlegel, G. E. Scuseria, M. A. Robb, J. R. Cheeseman, G. Scalmani, V. Barone, B. Mennucci, G. A. Petersson, H. Nakatsuji, M. Caricato, X. Li, H. P. Hratchian, A. F. Izmaylov, J. Bloino, G. Zheng, J. L. Sonnenberg, M. Hada, M. Ehara, K. Toyota, R. Fukuda, J. Hasegawa, M. Ishida, T. Nakajima, Y. Honda, O. Kitao, H. Nakai, T. Vreven, J. A. Montgomery Jr., J. E. Peralta, F. Ogliaro, M. Bearpark, J. J. Heyd, E. Brothers, K. N. Kudin, V. N. Staroverov, T. Keith, R. Kobayashi, J. Normand, K. Raghavachari, A. Rendell, J. C. Burant, S. S. Iyengar, J. Tomasi, M. Cossi, N. Rega, J. M. Millam, M. Klene, J. E. Knox, J. B. Cross, V. Bakken, C. Adamo, J. Jaramillo, R. Gomperts, R. E. Stratmann, O. Yazyev, A. J. Austin, R. Cammi, C. Pomelli, J. W. Ochterski, R. L. Martin, K. Morokuma, V. G. Zakrzewski, G. A. Voth, P. Salvador, J. J. Dannenberg, S. Dapprich, A. D. Daniels, O. Farkas, J. B. Foresman, J. V. Ortiz, J. Cioslowski, D. J. Fox, Gaussian, Inc., Wallingford CT, **2016**.

52 F. Neese, *WIREs Comput. Mol. Sci.* **2018**, *8*, e1327.

53 J. P. Perdew, K. Burke, M. Ernzerhof, *Phys. Rev. Lett.* **1996**, *77*, 3865.

54 J. P. Perdew, K. Burke, M. Ernzerhof, *Phys. Rev. Lett.* **1997**, *78*, 1396.

55 C. Adamo, V. Barone, *J. Chem. Phys.* **1999**, *110*, 6158.

56 S. Grimme, J. Antony, S. Ehrlich, H. Krieg, *J. Chem. Phys.* **2010**, *132*, 154104.

57 S. Grimme, S. Ehrlich, L. Goerigk, *J. Comput. Chem.* **2011**, *32*, 1456.

58 F. Weigend, R. Ahlrichs, *Phys. Chem. Chem. Phys.* **2005**, *7*, 3297.

59 C. Riplinger, F. Neese, *J. Chem. Phys.* **2013**, *138*, 034106.

60 D. G. Liakos, M. Sparta, M. K. Kesharwani, J. M. L. Martin, F. Neese, *J. Chem. Theory Comput.* **2015**, *11*, 1525–1539.

61 C. Riplinger, P. Pinski, U. Becker, E. F. Valeev, F. Neese, *J. Chem. Phys.*, **2016**, *144*, 024109.

62 C. J. Cramer, *Essentials of Computational Chemistry: Theories and Models*, John Wiley & Sons, Ltd, Chichester, UK, **2004**.

63 A. V. Marenich, C. J. Cramer, D. G. Truhlar, *J. Phys. Chem. B*, **2009**, *113*, 6378.
